# Spatial Clustering by Red Deer and Its Relevance for Management of Chronic Wasting Disease

**DOI:** 10.3390/ani11051272

**Published:** 2021-04-28

**Authors:** Atle Mysterud, Isa Nergård Skjelbostad, Inger Maren Rivrud, Øystein Brekkum, Erling L. Meisingset

**Affiliations:** 1 Centre for Ecological and Evolutionary Synthesis (CEES), Department of Biosciences, University of Oslo, P.O. Box 1066 Blindern, NO-0316 Oslo, Norway; i.skjelbostad@hotmail.com; 2 Norwegian Institute for Nature Research (NINA), Sognsveien 68, NO-0855 Oslo, Norway; inger.rivrud@nina.no; 3 Department of Forestry and Forestry Resources, Norwegian Institute of Bioeconomy Research, Tingvoll gard, NO-6630 Tingvoll, Norway; oystein.brekkum@nibio.no (Ø.B.); erling.meisingset@nibio.no (E.L.M.)

**Keywords:** agriculture, chronic wasting disease, hay bales, GPS, Norway, red deer, spatial clustering, wildlife disease

## Abstract

**Simple Summary:**

Chronic Wasting Disease is a deadly infectious disease affecting cervids that was discovered in Norway in 2016. CWD can transmit through environmental reservoirs and aggregation and spatial clustering of animals may affect transmission. Deer usually forage on scattered forage, but anthropogenic food sources are often concentrated in space, leading to spatial aggregation. We determined what caused red deer to revisit the same locations in the environment, and the extent to which this was caused by anthropogenic food sources. We document that the most visited sites were indeed anthropogenic, which opens potential avenues to disease mitigation.

**Abstract:**

Herbivores like cervids usually graze on widely scattered forage, but anthropogenic food sources may cause spatial revisitation and aggregation, posing a risk for transmission of infectious diseases. In 2016, chronic wasting disease (CWD) was first detected in Norway. A legal regulation to ban supplemental feeding of cervids and to fence stored hay bales was implemented to lower aggregation of cervids. Knowledge of further patterns and causes of spatial revisitation can inform disease management. We used a recently developed revisitation analysis on GPS-positions from 13 red deer (*Cervus elaphus*) to identify the pattern of spatial clustering, and we visited 185 spatial clusters during winter to identify the causes of clustering. Anthropogenic food sources were found in 11.9% of spatial clusters, which represented 31.0% of the clusters in agricultural fields. Dumping of silage and hay bales were the main anthropogenic food sources (apart from agricultural fields), and unfenced hay bales were available despite the regulation. The probability of the clusters being in agricultural fields was high during winter. It may be necessary to find other ways of disposing of silage and enforcing the requirement of fencing around hay bales to ensure compliance, in particular during winters with deep snow.

## 1. Introduction

Space use by large animals is a well-studied topic, partly due to advanced tracking technology such as Global Positioning Systems (GPS) [1]. Nevertheless, we know little about drivers and patterns of revisitation of animals to specific sites [2]. Such revisitation is sometimes used to infer, regarding memory and familiarity as playing a role for habitat selection [3], but revisitation may also arise due to unaccounted habitat heterogeneity [4]. Clustering of animals and frequent revisitation at specific locations may cause risk of transmission of infectious diseases. Large herbivores are adapted to feed on natural forage that is widely dispersed. However, during winter cervids can be quite opportunistic and often enter human dominated landscapes in search for food, and it is common to provide supplemental forage aimed at cervids [5]. Supplementary winter feeding has been shown to restrict movements of roe deer (*Capreolus capreolus*) [6] and moose (*Alces alces*) [7], and aggregation at feeding sites is a known risk factor for disease transmission [8]. Understanding patterns and drivers of spatial clustering is hence an important issue for disease management, as the extent to which anthropogenic sources cause such aggregation provides a potential avenue for disease mitigation.

Chronic wasting disease (CWD) is a lethal and contagious disease of cervids spreading across USA and Canada [9], and was recently discovered in Norway [10]. The prions causing CWD can transmit from animal-to-animal via direct contact or indirectly via environmental contamination [9]. Hence, any attraction points such as supplemental feeding sites and mineral licks are regarded as hot spots for transmission [11,12]. CWD was first registered in Norway in 2016 among wild reindeer (*Rangifer tarandus*) [10]. The first CWD infected population in Nordfjella was eradicated [13,14], but sympatric red deer (*Cervus elaphus*) have overlapping summer range with the area used by the infected reindeer population. Red deer are susceptible to CWD [15], and spillover of CWD to red deer is regarded an important threat by the Norwegian Scientific Committee for Food and Environment [16]. Due to CWD emergence, a national ban on winter feeding aimed at cervids was implemented in Norway a short time after [17], but we have no knowledge of whether other factors can lead to spatial clustering of red deer in the area.

**Figure 1:**
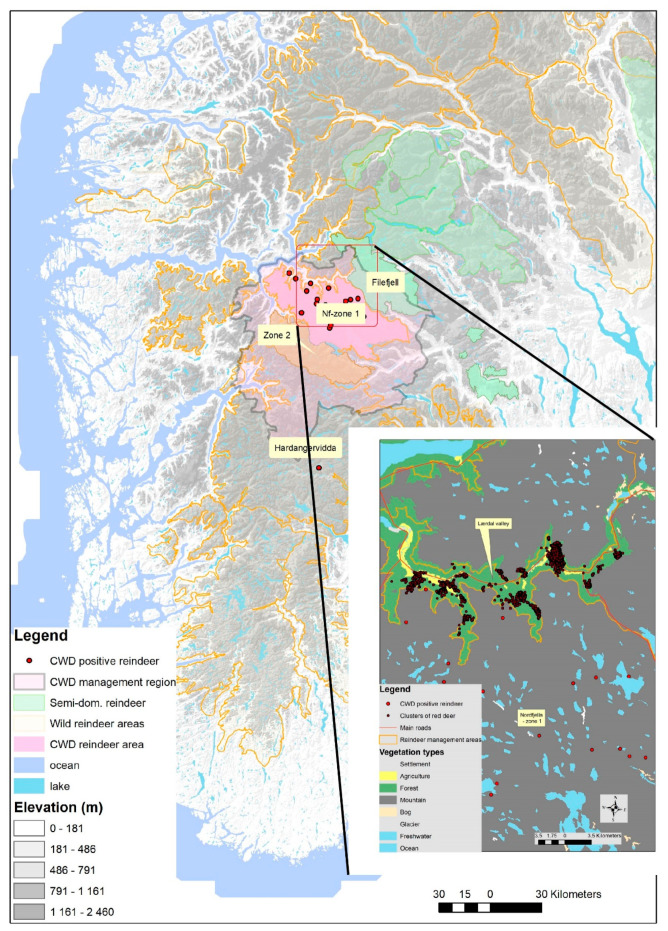
An overview of the study area in the Lærdal municipality in the Nordfjella region, Norway. (insert) Detailed positions of the winter clusters being visited in the field.

We here provide the first systematic analysis of patterns and drivers of spatial revisitation of cervids. As background, we use red deer in the Nordfjella region of Norway due to the imminent threat of CWD spillover from reindeer to red deer. The whole Nordfjella region is included in a CWD management zone, where red deer populations are actively managed to lower risk of CWD spillover and transmission (Figure 1). Classical CWD is not yet detected in red deer, but in the population of reindeer with spatial overlap with red deer (Figure 1). Knowledge about potential transmission hot spots would enable improved management of CWD transmission risk among red deer if spillover from reindeer occurs. Our overall aim is to better understand drivers and patterns of red deer spatial clustering and aggregation as a basis for management of CWD. This is also made relevant with the recent (September 2020) detection of CWD in reindeer in a new area, the Hardangervidda region [18]. Our study consists of two parts; (1) analysis of patterns of spatial revisitation of 13 GPS-collared red deer through the annual cycle (2017–2019), and (2) field visits to revisitation sites during winter 2019 to determine likely drivers of spatial clustering behavior of these red deer in the Lærdal municipality with the densest red deer population in the Nordfjella region of Norway. We quantified specifically the proportion of spatial clusters containing anthropogenic sources of food, e.g., leftover silage or feeding stations placed for livestock, as this may provide a basis for disease mitigation.

## 2. Materials and Methods

### 2.1. Ethics Statement

Red deer were marked during winters following standard protocols [19] and approved by the Norwegian Food Safety Authority (FOTS ID 19113) and the Norwegian Environment Agency.

### 2.2. Study Area

The study area comprises the Nordfjella region, Norway (Figure 1). The area covers typical fjord areas with dense red deer populations in the Aurland and Lærdal municipality in Vestland county in the west, to more inland areas with lower density of red deer in the east. The western part of the region is characterized by varied topography dominated by alpine mountain landscape with valleys and fjords. Our focal area Lærdal municipality has a 51 km long main valley where red deer aggregate during winter. The bottom of the valley consists of agricultural fields, roads, and buildings. The slopes leading down to the valley from the mountains are covered mainly with deciduous forest, birch (*Betula* sp.) and alder (*Alnus incana*), with some scattered Scots pine (*Pinus sylvestris*) and Norway spruce (*Picea abies*). These deciduous forests towards the mountains are summer ranges for red deer and overlapping with the area used by the previously CWD infected reindeer population. The mean temperature for January and February 2019 in the bottom of the valley was −0.2 °C, and the mean precipitation in this period was 1.3 mm/day.

### 2.3. Study Design and Field Work

We marked 13 red deer yielding data for 2017–2019 in the Lærdal municipality of Norway. Lærdal have the highest density of red deer (~5 per km^2^ during winter) and the most extensive spatial overlap with the area used by the CWD infected reindeer population. GPS collars (VECTRONIC Aerospace GmbH, Berlin, Germany) with an integrated VHF-transmitter were set to take positions every hour.

The field work was conducted from 7th to 11th of January and from 4th to 17th of February 2019. Before each period of field work, the most recent GPS-positions were used to define spatial clusters for each individual. We used revisitation analysis to identify the number and position of clusters for a given definition [2]. We defined clusters as areas of 20 m^2^ that the animal had visited 3 times or more during a 14 day period prior to field work, i.e., when a minimum of 3 GPS-positions were within a circle of 20 m^2^. For the later analysis of the full annual pattern, we used a lower threshold of 5 GPS-positions to define a cluster, due to a longer period of observation (month). We calculated the number of clusters per individual and month, and how many positions a given cluster consisted of.

Due to logistic constraints, there was a trade-off between full randomization and efficient sampling in these extreme mountainous landscapes with steep slopes and few roads. The focus was to get as many of the clusters from sites around human settlements and agricultural areas, because potential hotspots for disease transmission in agricultural fields were more accessible and more likely to be anthropogenic. There was nevertheless gathered a sufficient sample from forest habitats to get a more complete picture of the drivers of clustering. From the full GPS-data set, we later estimated how this sampling biased estimates of clustering in agricultural fields versus forest.

### 2.4. Field Site Descriptors of Habitat

The following habitat variables were described or measured both in the center of each spatial cluster and in a paired random location in a randomly selected direction and distance between 50–100 m away from the spatial cluster:

- Agricultural fields vs. forest. The type of agricultural field was further categorized as either meadow or another form of cultivated land (other).
- Coverage (%) of grasses.
- Canopy cover (%) was measured by using a spherical densiometer [20].
- Distance to nearest tree (m) over 2 m tall was measured using a binocular with range finder (Leica Geovid 10 × 40) or using strides for shorter distances.
- Aspect (0 to 400) and slope (in °) was measured using a handheld compass.
- Distance to nearest (visible) saltlick, hay bales and feeding station. We noted whether hay bales were fenced, fenced with open gate, or not fenced.
- Counts of feces, bed sites and tracks. We counted the number of feces dung, bedding sites and tracks of cervids and livestock within a 5 m radius whenever tracking conditions allowed.

### 2.5. Broad Habitat Categories

In line with our previous work on habitat selection of red deer [21,22], we used the AR50 map provided by the Norwegian Institute for Bioeconomy (online open: kilden.nibio.no). We combined all agricultural fields (habitat type 20), while forest (type 30), bog (type 60), and naturally open areas (type 50) were categorized as outfield, but for simplicity referred to as forest, which was the dominant category. The few clusters in freshwater (type 81) likely arose from measuring inaccuracy and were removed.

### 2.6. Statistical Analyses

To analyze a local selection of sites, we used a paired t-test to compare each spatial cluster against the paired random site for all habitat variables. This was done separately for sites in agricultural fields and in forest. Data measured as percentage were arcsin-square root transformed.

We analyzed variation in spatial position of clusters relative to elevation (m), use of agricultural fields (field vs. forest), and in number of clusters per individual. We initially used a generalized additive model (GAM) plot in library ‘mgcv’ to determine non-linear patterns [23]. We plotted elevation against Julian date and found that four categories provided a fair description of the seasonal pattern; i.e., winter (January to April), spring (May), summer (June to August) and fall (September to December). Our main analyses were linear mixed effects models fitted in R vs. 3.6.2 using the ‘lme4′ library [24]. We used individual ID as a random term. We considered the category year (2017, 2018 and 2019), season (winter, spring, summer and fall) and the interaction term as fixed effects. As we did not have all seasons in 2019, we fitted the interaction using a dummy variable combining year and season. We used Akaike Information Criterion (AIC) for backwards model selection. The final model was fitted using a bootstrap with 100 replicates to calculate confidence limits of estimates and predicted values. To look at the effect of snow depth on elevation of clusters, the analysis was subsequently limited to the months January–March, which had a noticeable amount of snow.

For use of agricultural fields (field/forest), we used generalized linear mixed effects models with a binomial distribution and with the same random term and fixed effects.

For the number of spatial clusters per individual and month, we used a negative binomial model within the glmmTMB package. All analyses were run in R vs. 4.0.3.

## 3. Results

### 3.1. Winter Field Data

We visited in total 185 spatial clusters in the field during winter 2019. Of these, 31.9% (*n* = 59) were located in agricultural fields, and the other 68.1% (*n* = 126) were located in forest. Anthropogenic feeding was found in 11.9% (*n* = 22) of spatial clusters, which represent 30.5% (*n* = 18) of the clusters in agricultural fields. One of these clusters had a salt lick. None of the spatial clusters had hay bales within the cluster site itself, but hay bales were within sight for 11.9% (*n* = 7) of the agricultural field clusters. In total, 6.7% (*n* = 4) of the field clusters were near hay bales with no fence (all observation on the same site), 3.3% (*n* = 2) were near hay bales with an open fence (one site), and 1.6% (*n* = 1) were near hay bales that had been fenced in. There was a significantly higher number of red deer tracks, feces dung and bedding sites in spatial clusters compared to nearby random sites. Agricultural field clusters had more tracks of deer than clusters in the forest (Table 1). Roe deer tracks were only found in 4.3% of the in total 370 sites visited, while no moose, reindeer, cattle or sheep tracks were found.

### 3.2. Annual Pattern

**Figure 2:**
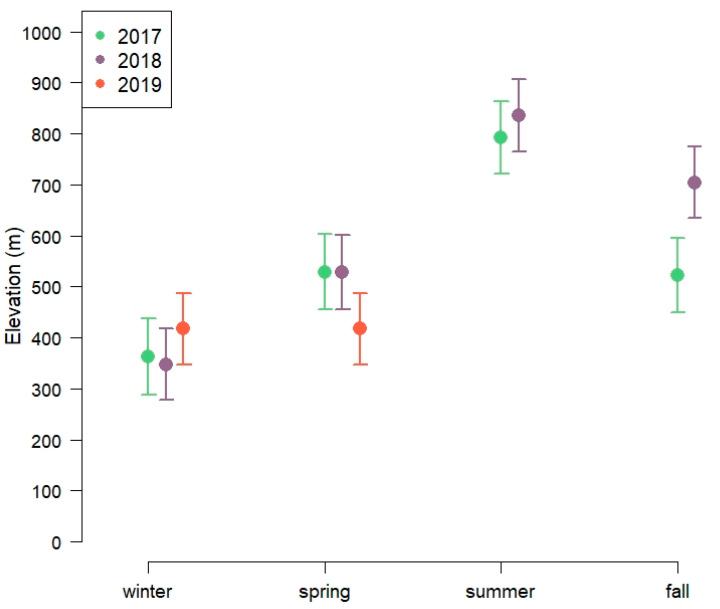
Elevational position of spatial clusters during different seasons and years of 13 GPS-marked red deer in Lærdal municipality, Norway.

*Elevation.* The pattern of elevation use was best described by the full model with season, year and their interaction (Table S1). The clusters were at lower elevations (~300–500 m above sea level) during the winter season, highest during summer months (~700–900 m) with spring (~400–600 m) and fall being intermediate (~500–800 m; Figure 2, Table S2). For the winter months (January–March), the full model with year, snow depth and their interaction had lowest AIC (Table S3). The estimated effects of snow depth on elevation were stronger in 2019 than in 2018 (Table S4).

**Figure 3:**
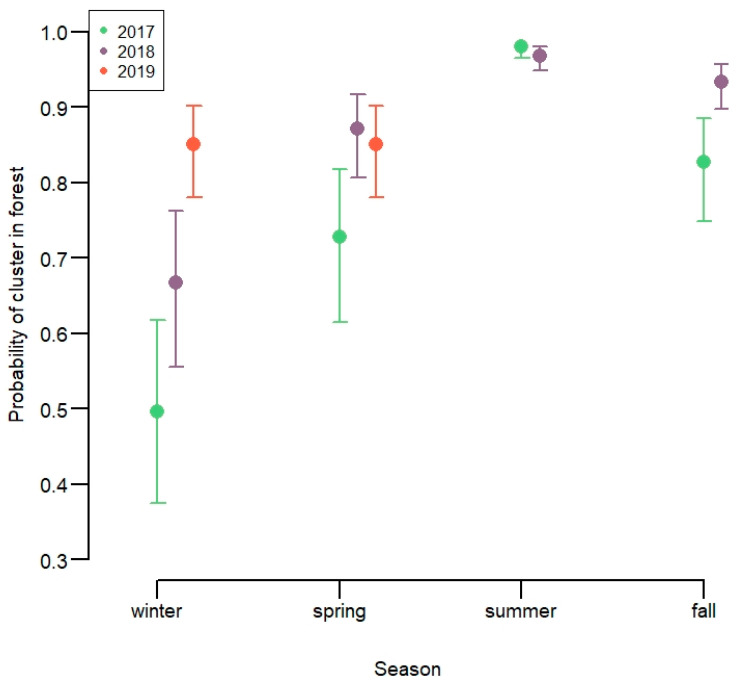
Proportion of clusters in forest compared to on agricultural fields of 13 GPS-marked red deer in Lærdal municipality, Norway.

*Agricultural fields*. Overall, 16.7% of clusters were in agricultural fields (*n* = 39,334), and for the winter season, 39%. The probability of clusters being in agricultural fields varied depending on the interaction between season and year (Figure 3, Tables S1 and S2). There was low probability of clusters being on agricultural fields during summer in both 2018 and 2019. The probability of clusters being on agricultural fields during the winter varied strongly depending on year, from around 0.5 in 2017 to 0.15 in 2019 with 2018 being intermediate. Spring and fall months had intermediate probability of being on agricultural fields. The probability of the clusters being in agricultural fields decreased with an increasing amount of snow (Table S4).

**Figure 4:**
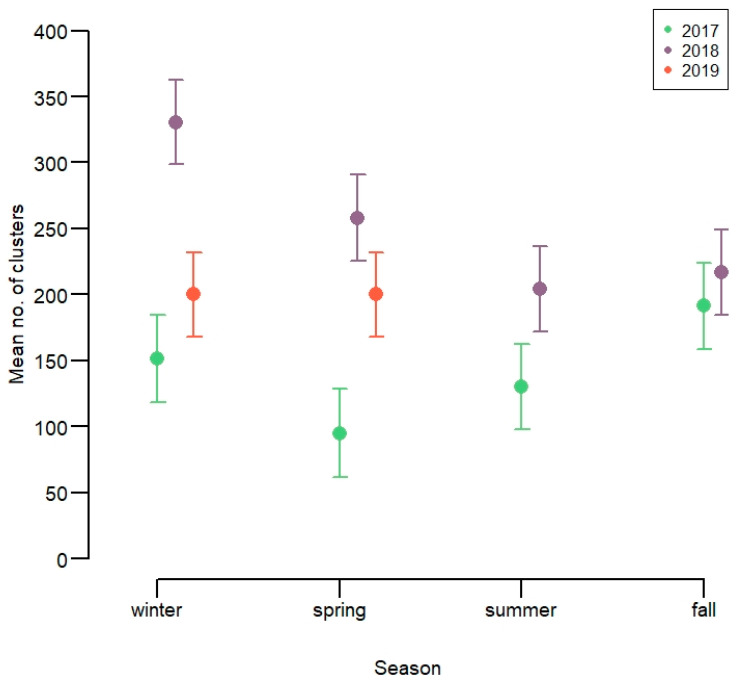
Number of spatial clusters of 13 GPS-marked red deer in Lærdal municipality, Norway.

*Number of spatial clusters*. The average monthly number of spatial clusters per animal varied strongly from year to year, but less markedly across seasons (Figure 4, Tables S1 and S2). There were fewer clusters per animal per month in 2017 (150–200). In 2018, there were more clusters per animal per month in winter and spring (250–350), compared to summer and fall (200–250). In 2019, the average number of clusters was intermediate. The average number of spatial clusters per month and individual increased with increasing snow depth only in 2018 (Table S4), which is likely also the cause of more winter clusters this year.

**Figure 5:**
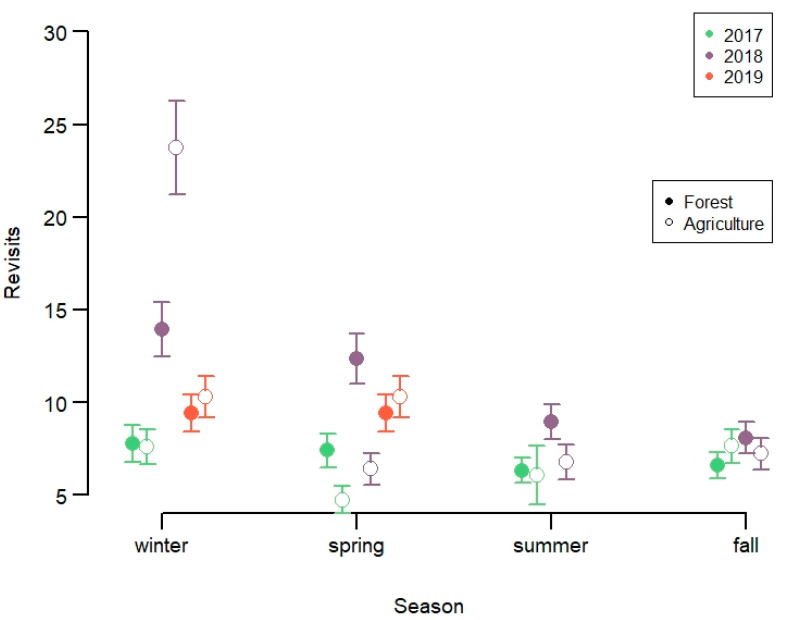
Number of GPS-positions per spatial cluster of marked red deer in Lærdal municipality, Norway. Note that minimum number of revisits was 5 as of definition.

*Number of revisits*. The number of positions within a cluster averaged 12 GPS-positions (given the 5 GPS-positions threshold), but was up to 93 positions. Only 2.9% of clusters had more than 50 GPS-positions, but this represented 8.3% of clusters in agricultural fields. The total number of fixes per individual and month was on average 696 and with a maximum possible of 744 for months with 31 days. The number of revisits was best explained by the interaction term between year, season and habitat (Table S5). The number of revisits was higher in agricultural fields than in forest, and was much higher in 2018 than the other years, in particular during winter (Figure 5, Table S6).

## 4. Discussion

The recent outbreak of CWD in Norway makes it urgent for wildlife management to understand what factors cause cervids to aggregate and revisit the same sites. In human dominated landscapes, it is most relevant to identify anthropogenic drivers of spatial clustering, as they provide a key to disease mitigation. A total of 16.7% of clusters were in agricultural fields. The highest probability of clustering on agricultural fields was found during winter, but there were large annual variations. Overall, 11.9% of clusters contained anthropogenic supplements, which accounts for 31% of the spatial clusters in agricultural fields. This yields some potential for disease mitigation, pointing in particular to targeting periods with deep snow when red deer gather at low elevation.

### 4.1. Pattern of Spatial Clustering

Habitat selection results from choices at different scales [25,26]. The position of clusters will reflect both the position of the home range during the annual cycle and within seasonal home range behavior causing the actual revisitation. Pattern of spatial clustering with regard to elevation followed largely what was expected for this partially migratory population [27], with high elevation summer ranges and low elevation winter ranges consistently from year to year. Agricultural fields are in the valley bottoms, and are therefore more commonly used during fall, winter and spring when red deer gather in the valley as part of their winter range [21]. A surprising result was the lack of a clear seasonal pattern in the number of clusters per animal, while there was considerable annual variation in particular for winter and spring. The year 2018 was snow rich compared to normal winter conditions, while there was only shallow snow during winter of 2019. The highest number of clusters per animal and the high number of revisits to particular spots were in 2018 likely resulting from the deep snow. As snow depth increases, forage in agricultural areas becomes less accessible. This may explain why there was more clustering in forest habitat that is in steep terrain in this area, and the slightly surprising result of increasing elevation with increasing snow reflecting this spatial dislocation.

### 4.2. Causes of Clustering during Winter

Foraging by large herbivores in northern environments is markedly different during the growing season versus the non-growing season. Forage is continuously renewed and of high quality during the growing season, while herbivores forage on a depleting resource of lower quality during winter [28]. Winter is therefore a critical period for cervids due to low abundance and quality of food [29], and this also leads to use of anthropogenic food sources [30]. Intended supplemental feeding is very common at northern latitudes, but we know much less about the opportunistic use of other anthropogenic food sources. As part of disease mitigation by the Norwegian Food Safety Authority, all anthropogenic feeding of cervids is banned by a legal regulation [31]. Whether the observed anthropogenic food was intentional or non-intentional was difficult to determine, as compliance to the ban has been variable [17].

**Figure 6:**
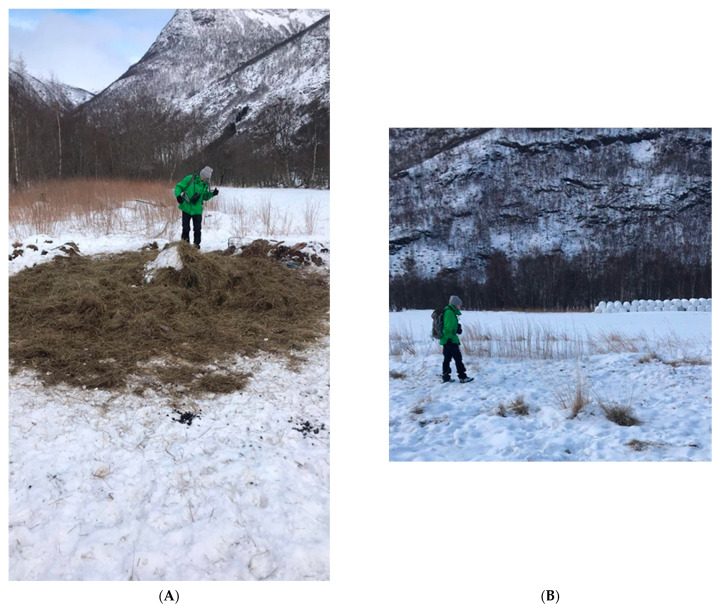
(**A**) The main driver of spatial clustering on agricultural fields was leftover silage from livestock farming. (**B**) Hay bales stored in on agricultural fields without being fenced.

Our field work was deliberately biased towards habitat close to humans, yielding 31.9% of the spatial clusters in agricultural fields visited during the field work in comparison to 20% as quantified by the unbiased GPS-data for the same winter and year. We found anthropogenic food sources at 11.9% of the clusters visited during the field work. The anthropogenic food consisted of silage dumped or hay bales in or around the edge of fields, which were not fenced as they were supposed to be (Figure 6). The remaining two sources (out of 22) were potatoes dumped in large quantities at the edge of a small field and supplemental feeding intended for cattle. Untouched hay bales could be seen from 11.9% of the agricultural field clusters. Anecdotal evidence from speaking to farmers suggest clustering at hay bales was more of a problem during the severe winter of 2017/18. With deep snow, the winter range of the red deer population also contracts to a smaller area leading to a higher effective population density, potentially further increasing the risk of contact between individuals. Leftover silage was clearly the dominant type of anthropogenic food during the field studies of winter 2018/19, with little snow. Implementing other ways to dispose of silage may reduce the aggregation on agricultural fields.

### 4.3. Clustering Versus Aggregation

The number of clusters will clearly be a matter of definition. We chose to use a fairly small number of positions for revisitation. This was due to our setting of using two weeks data of positions taken every hour to identify causes of recent clustering during in-field visits. Clearly, one may consider using more positions when identifying clusters with the highest impact on disease transmission risk, and also grade the importance of clusters depending on the number of visits and for how long duration they appear as attraction points. We rather chose to analyze the pattern of revisits independently (Figure 5). As much as 8.3% of the clusters in agricultural habitat consisted of >50 GPS-positions, and such clusters provide a natural starting point for mitigation. Further, spatial clustering by single individuals is not necessarily identical to contact points between individuals required to transmit pathogens. Red deer are social animals that stay in groups in these areas [32]. The GPS-marked red deer in our study were often in groups and tracks were observed from multiple deer at the clusters. Most spatial clusters were nevertheless occurring in forests, but these may not have the same relevance for disease management. Cervids spend their time alternating in bouts of ruminating and foraging during the winter [33]. A large proportion of the spatial clusters in the forest appeared to be bedding sites. Though frequently used, they are less likely to form an important part of CWD transmission, as if there is limited feeding, there will not be transfer of pathogens in environmental reservoirs. There were more tracks at spatial clusters in agricultural fields compared to forest. Hence, agricultural field clusters may play a larger role for disease transmission compared to their proportion of clusters from the total, supported by a higher number of GPS-positions per cluster and tracks from more individuals, indicative of aggregation.

Clustering in the agricultural landscape may also facilitate inter-species transmission, i.e., pathogen spillover. Roe deer are less migratory than red deer in the area [34] and generally across Europe [35]. Roe deer are more linked to the agricultural landscape year-round, and hence have less overlap with the previously CWD infected reindeer population. Infection in red deer will increase likelihood of spillover to roe deer as they share sympatric winter range. In 4.3% of the red deer spatial clusters, and in 10% of the agricultural field clusters, roe deer tracks were found. This shows that red deer and roe deer aggregate and forage on the same resources, increasing likelihood of disease spillover.

## 5. Conclusions

Factors causing aggregation on agricultural fields will be easier to manage than those causing aggregation in forest. We found that anthropogenic food sources were linked to agricultural fields or close to forest edge. Knowing more about the cause of aggregation and its anthropogenic sources before a disease outbreak could lead to more rapid and effective mitigation. It is well-known that red deer forage on hay bales if they get access. Finding ways to dispose of silage from livestock farms making it inaccessible to red deer and other cervids appears as the most immediate suggestion from our work. It is also critical with compliance to the measures already implemented. The Norwegian Food Safety Authority required all hay bales in Lærdal to be fenced in from 31st of March 2017 as part of the measures implemented to reduce the risk of CWD transmission. We noted some lack of compliance to this regulation. Assumedly the fences were left open for farmers to get easy access to the hay bales during the day, and farmers informed us that fences were closed the previous winter (2017/18) when deep snow attracted deer to the hay bales. They hence seem to have taken a pragmatic approach using the fences only under conditions when red deer approach them. Therefore, this issue appears to be particularly important during years with a lot of snow. Enforcement of the fencing requirement may be necessary during such periods.

## Figures and Tables

**Table 1:** Descriptive statistics (means) and t-tests comparing spatial clusters of red deer and paired random sites in agricultural fields and forest in Lærdal municipality, Norway, during winter 2019.

	Agricultural Fields				Forest			
Parameter	Cluster	Random	T	*p*	Cluster	Random	T	*p*
Canopy cover (%)	1.0	51.5	−9.580	<0.001	79.4	41.5	6.988	<0.001
Grass (%)	70.0	65.6	0.948	0.357	14.5	22.8	−1.390	0.181
Distance to tree (m)	31.1	5.3	4.405	<0.001	2.3	10.1	−4.713	<0.001
Slope (degrees)	6.5	19.5	−5.332	<0.001	24.1	19.8	2.746	0.007
Faeces	7.8	2.2	5.161	<0.001	6.6	2.4	6.925	<0.001
Bedding sites	0.4	0.1	2.939	0.005	0.6	0.0	5.664	<0.001
Tracks red deer	7.8	3.0	4.008	<0.001	3.6	2.9	1.624	0.110
Tracks roe deer	0.3	0.1	1.000	0.323	0.0	0.1	−1.371	0.176

## Data Availability

Data are available from authors on reasonable request.

## Supplementary Materials

The following are available online at https://www.mdpi.com/article/10.3390/ani11051272/s1, Table S1: Model selection variation in elevation, habitat, and number of clusters, Table S2: Parameter estimates variation in elevation, habitat, and number of clusters, Table S3: Model selection effect of snow depth, Table S4: Parameter estimates effect of snow depth, Table S5: Model selection number of revisits per cluster. Table S6: Parameter estimates number of revisits per cluster.
