# Supplementary material for: Spatial Clustering by Red Deer and Its Relevance for Management of Chronic Wasting Disease
Source: Animals (Basel). 2021 Apr 28;11(5):1272. doi: 10.3390/ani11051272 (PMC8146590; doi:10.3390/ani11051272)
Supplement: Supplementary file 1 [file animals-11-01272-s001.zip › animals-1194705-supplementary.pdf]

Supplementary Materials

Table S1. Model selection results using the Akaike Information Criterion (AIC) for all years (2017-19). Models compared were generalized linear mixed effects models with individual ID as random term and candidate fixed effects as listed. The response variable of the candidate models is shown in bold. ΔAIC is AIC relative to best model.

Year	Season	Season x year	AIC	ΔAIC
**Elevation**				
1			540403.3	27451.2
	1		515088	2135.9
1	1		514002.8	1050.7
1	1	1	512952.1	0
**Agriculture**				
1			29894	3968.6
	1		27208	1278.5
1	1		26129	199.5
1	1	1	25929.5	0
**Number of clusters**				
1			482778.8	3896.2
	1		486022.7	7140.1
1	1		480396.1	1486.5
1	1	1	478882.6	0

Table S2. Parameter estimates for generalized linear mixed effects models analysing elevation, habitat (forest vs. agricultural habitat), and number of clusters per individual for red deer in Lærdal, Norway 2017-19. Baseline for season and year was fall 2017. Individual ID is fitted as a random intercept with std. dev. = 143.5, 0.8742 and 67.89 respectively for elevation, habitat and cluster number.

Parameter	Estimate	SE	Lower 95% limit	Upper 95% limit
**Elevation**				
Intercept	522.748	40.180	443.995	601.501
Fall 2018	181.574	6.263	169.299	193.849
Spring 2017	6.372	12.081	-17.307	30.051
Spring 2018	5.490	8.264	-10.707	21.687
Spring 2019	-4.500	11.247	-26.544	17.544
Summer 2017	269.755	8.435	253.222	286.288
Summer 2018	312.498	6.543	299.674	325.322
Winter 2017	-159.866	10.759	-180.954	-138.778
Winter 2018	-174.907	6.127	-186.916	-162.898
Winter 2019	-105.851	6.607	-118.801	-92.901
**Habitat**				
Intercept	1.388	0.256	0.886	1.890
Fall 2018	1.137	0.105	0.931	1.343
Spring 2017	-0.345	0.167	-0.673	-0.018
Spring 2018	0.534	0.140	0.260	0.808
Spring 2019	1.011	0.224	0.572	1.449
Summer 2017	2.449	0.280	1.900	2.999
Summer 2018	1.968	0.138	1.697	2.239
Winter 2017	-1.338	0.138	-1.608	-1.068
Winter 2018	-0.495	0.088	-0.668	-0.322
Winter 2019	0.289	0.104	0.085	0.492
**Number of clusters**				
Intercept	191.185	18.955	154.033	228.337
Fall 2018	25.560	2.445	20.768	30.352
Spring 2017	-96.332	5.101	-106.330	-86.334
Spring 2018	66.922	3.147	60.754	73.090
Spring 2019	-94.674	4.675	-103.837	-85.511
Summer 2017	-61.224	3.394	-67.876	-54.572
Summer 2018	13.175	2.573	8.132	18.218
Winter 2017	-39.703	4.455	-48.435	-30.971
Winter 2018	139.396	2.299	134.890	143.902
Winter 2019	8.661	2.593	3.579	13.743

Table S3. Model selection results using the Akaike Information Criterion for subset of winter months (January-March) related to snow depth (2018-19). Models compared were generalized linear mixed effects models with individual ID as random term and candidate fixed effects as listed. The response variable of the candidate models is shown in bold.

Year	Snow depth	Snow depth x year	AIC	ΔAIC
**Elevation**				
1			48617.0	109.1
	1		49431.1	923.2
1	1		48538.9	31.1
1	1	1	48507.8	0
**Agriculture**				
1			3813.4	20.1
	1		3908.2	114.8
1	1		3793.4	0
1	1	1	3795.0	1.6
**Number of clusters**				
1			145680.8	1484.1
	1		146321.6	2124.9
1	1		144323.1	126.4
1	1	1	144196.7	0

Table S4. Parameter estimates for generalized linear mixed effects models analysing the effect of snow depth (in mm and scaled) on use of elevation, habitat (forest vs. agricultural habitat), and number of clusters per individual for red deer during winter in Lærdal, Norway 2018-19. Baseline for year was 2018. Individual ID is fitted as a random intercept with std. dev. = 177.9, 1.091 and 82.04 respectively for elevation, habitat and cluster number.

Parameter	Estimate	SE	Lower 95% limit	Upper 95% limit
**Elevation**				
Intercept	324.35	49.40	227.52	421.17
Year 2019	100.31	3.14	94.15	106.46
Snow depth	9.96	2.03	5.98	13.93
Snow depth x Year 2019	20.12	3.76	12.75	27.48
**Habitat**				
Intercept	0.835	0.311	0.226	1.444
Year 2019	1.125	0.106	0.917	1.333
Snow depth	0.286	0.062	0.165	0.407
**Number of clusters**				
Intercept	321.70	22.79	277.03	366.37
Year 2019	-101.13	2.11	-105.27	-96.99
Snow depth	48.27	1.24	45.84	50.70
Snow depth x Year 2019	-28.28	2.53	-33.23	-23.33

Table S5. Model selection results using the Akaike Information Criterion for all years (2017-19) for the response variable "number of revisits". Models compared were generalized linear mixed effects models with individual ID as random term and candidate fixed effects as listed. The "season x year" interaction term was a dummy variable due to no data from summer and fall 2019 (see methods), and therefore only habitat interaction with "season x year" was run.

Year	Season	Season x year	Habitat	Habitat:season:year	AIC	ΔAIC
1					251740.0	8747.1
1	1				245677.1	2684.0
1	1	1			245199.8	2206.7
1	1	1	1		244399.1	1406.0
1	1	1	1	1	242993.1	0

Table S6. Parameter estimates for a generalized linear mixed effects model analysing number of revisits per cluster for red deer in Lærdal, Norway 2017-19. Baseline for season and year was fall 2017. Individual ID is fitted as a random intercept with std. dev = 0.1913.

Parameter	Estimate	SE	Lower 95% limit	Upper 95% limit
Intercept	2.033	0.060	1.915	2.151
Fall 2018	-0.057	0.038	-0.132	0.018
Spring 2017	-0.477	0.065	-0.604	-0.351
Spring 2018	-0.176	0.050	-0.275	-0.077
Spring 2019	-0.227	0.095	-0.413	-0.042
Summer 2017	-0.231	0.127	-0.480	0.018
Summer 2018	-0.117	0.054	-0.222	-0.011
Winter 2017	-0.007	0.044	-0.093	0.079
Winter 2018	1.135	0.030	1.076	1.193
Winter 2019	0.298	0.035	0.230	0.367
Habitat (forest vs. agriculture)	-0.146	0.031	-0.207	-0.085
Habitat x Fall 2018	0.260	0.041	0.180	0.341
Habitat x Spring 2017	0.591	0.074	0.446	0.737
Habitat x Spring 2018	0.802	0.054	0.696	0.908
Habitat x Spring 2019	0.234	0.099	0.040	0.428
Habitat x Summer 2017	0.190	0.129	-0.063	0.442
Habitat x Summer 2018	0.422	0.056	0.312	0.532
Habitat x Winter 2017	0.172	0.059	0.057	0.288
Habitat x Winter 2018	-0.386	0.034	-0.452	-0.320
Habitat x Winter 2019	0.056	0.038	-0.019	0.130
